# OCT angiography of persistent hyaloid artery: a case report

**DOI:** 10.1186/s12886-019-1155-5

**Published:** 2019-07-04

**Authors:** Hansol Jeon, Jinsoo Kim, Soonil Kwon

**Affiliations:** Department of Ophthalmology, Hallym University College of Medicine, Hallym University Sacred Heart Hospital, 22, Gwanpyeong-ro 170beon-gil, Dongan-gu, Anyang, 14068 South Korea

**Keywords:** Persistent hyaloid artery, Hyaloid artery, Vitreous hemorrhage, Case report

## Abstract

**Background:**

A persistent hyaloid artery is a rare fetal remnant. Several complications such as amblyopia, vitreous hemorrhage, and retinal detachment have been reported. Here, we present a case of vitreous hemorrhage with a persistent hyaloid artery.

**Case presentation:**

A healthy 16-year-old male presented with blurred vision in his left eye. Vitreous hemorrhage occurred and absorbed spontaneously. Slit-lamp examination demonstrated a *Mittendorf’s dot* and fundus examination revealed a persistent hyaloid artery. Optical coherence tomography (OCT) showed a *Bergmeister’s papilla.* The blood flow of the persistent hyaloid artery via the *Bergmeister’s papilla* was found by OCT angiography.

**Conclusion:**

The persistent hyaloid artery should be considered as a cause of spontaneous vitreous hemorrhage of young healthy patient. The OCT angiography will be a useful noninvasive approach to confirm the patency of the persistent hyaloid artery.

## Background

The process of intraocular vascularization begins with the entry of the hyaloid artery into the optic disc cup through the fetal fissure. Under normal conditions, the artery atrophies at its midpoint and retracts to its end at the optic disc and the posterior pole of lens at term [1].

A persistent hyaloid artery (PHA) results from the failure of apoptosis of hyaloid vascular system. Despite numerous growth factors and molecules implicated on few studies, factors associated to apoptosis of the hyaloid artery are still unknown [2]. The PHA is associated with rare but severe vitreous hemorrhage (VH), retinal detachment and cataract [2–8].

In previous reports, fundus photography, fluorescein angiography (FAG), optical coherence tomography (OCT) and Doppler ultrasound were used to diagnose and to check the blood flow of the PHA [3–6, 8]. OCT angiography can visualize peripapillary vessels by detecting the movement of red-blood cells [9]. Herein, we present the case of VH from the PHA which was confirmed by OCT angiography.

## Case presentation

A healthy 16-year-old Korean male presented to our clinic in September 26, 2018 with blurred vision in his left eye. He had neither past medical history nor trauma history.

**Fig. 1:**
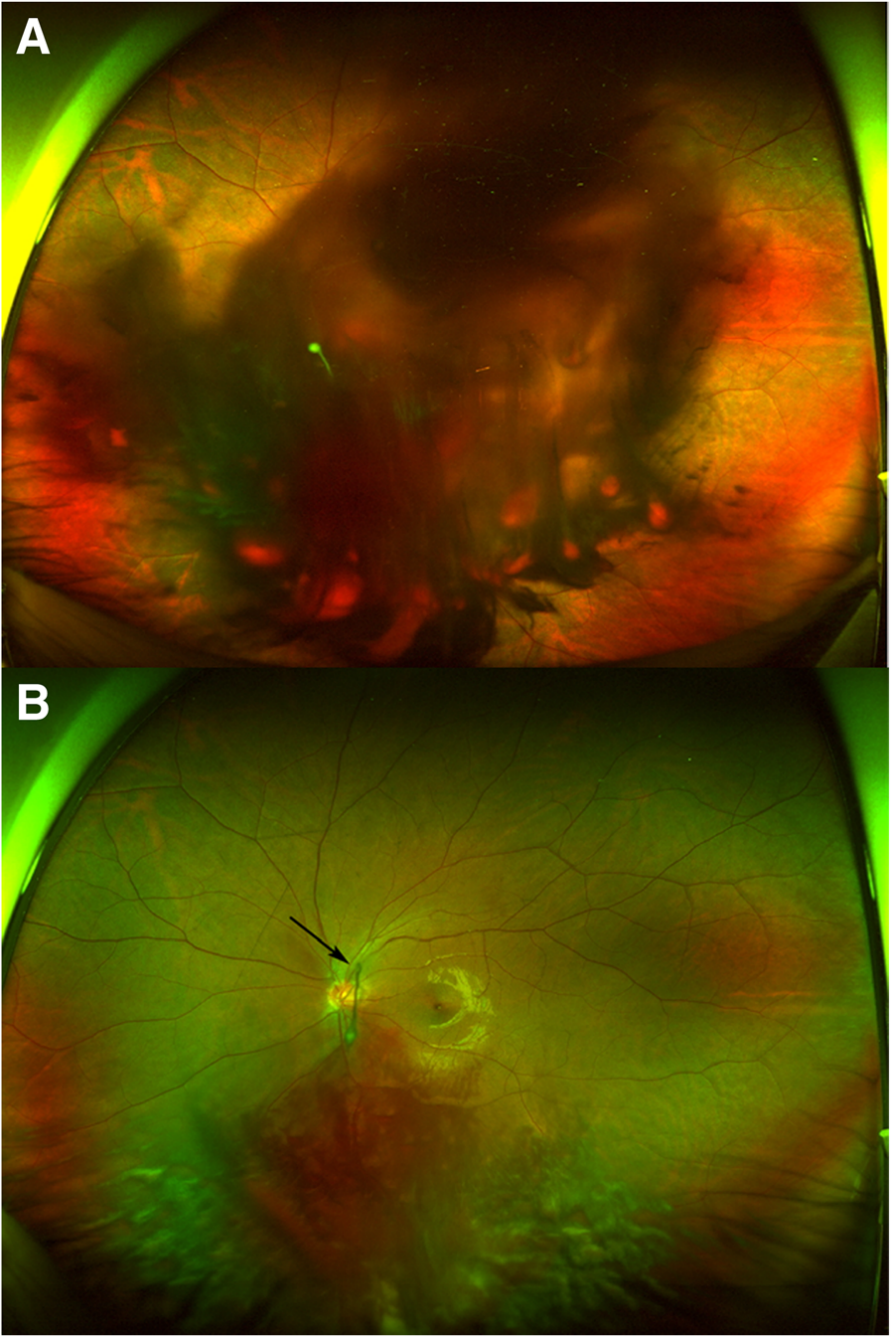
Wide field fundus photography of the patient (**a**) Massive vitreous hemorrhage was seen at the initial visit. **b** Vitreous hemorrhage was partially absorbed after 2 weeks. The persistent hyaloid artery was seen (arrow)

On ocular examination, best corrected visual acuity (BCVA) was 0 logMAR in his right eye and Hand motion in his left eye. Intraocular pressures were 17 mmHg in the right eye and 13 mmHg in the left eye. The fundus was invisible due to massive VH in the left eye (Fig. 1a). His blood pressure and laboratory test results including coagulating factors were normal.

**Fig. 2:**
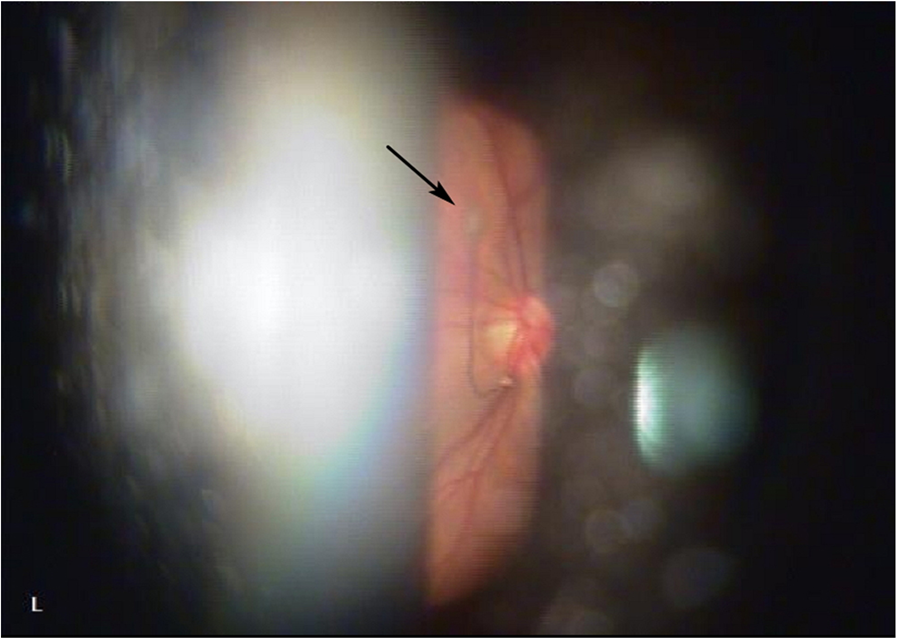
Slit-lamp photography was taken at 2 weeks after the initial visit. The point of insertion of the persistent hyaloid artery on the inferior nasal quadrant of the posterior lens capsule (*Mittendorf’s dot*) is indicated by the arrow

**Fig. 3:**
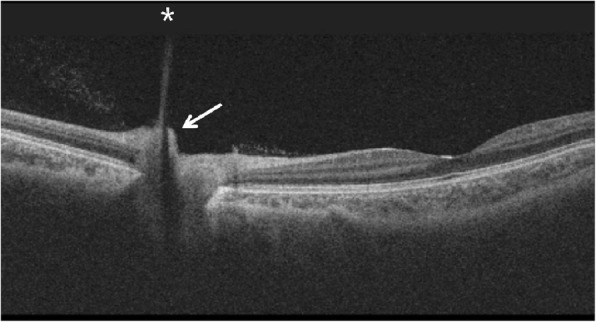
Optical coherence tomography of optic nerve of the left eye of the patient taken at 2 weeks after the initial visit reveals the posterior aspect of the persistent hyaloid artery. OCT shows hyporeflective tubular structure of the persistent hyaloid artery (asterisk) and an elevated tissue structure (*Bergmeister’s papilla)* (arrow). (Swept Source DRI OCT TRITON. Topcon Medical Systems, Inc. Oakland, USA)

**Fig. 4:**
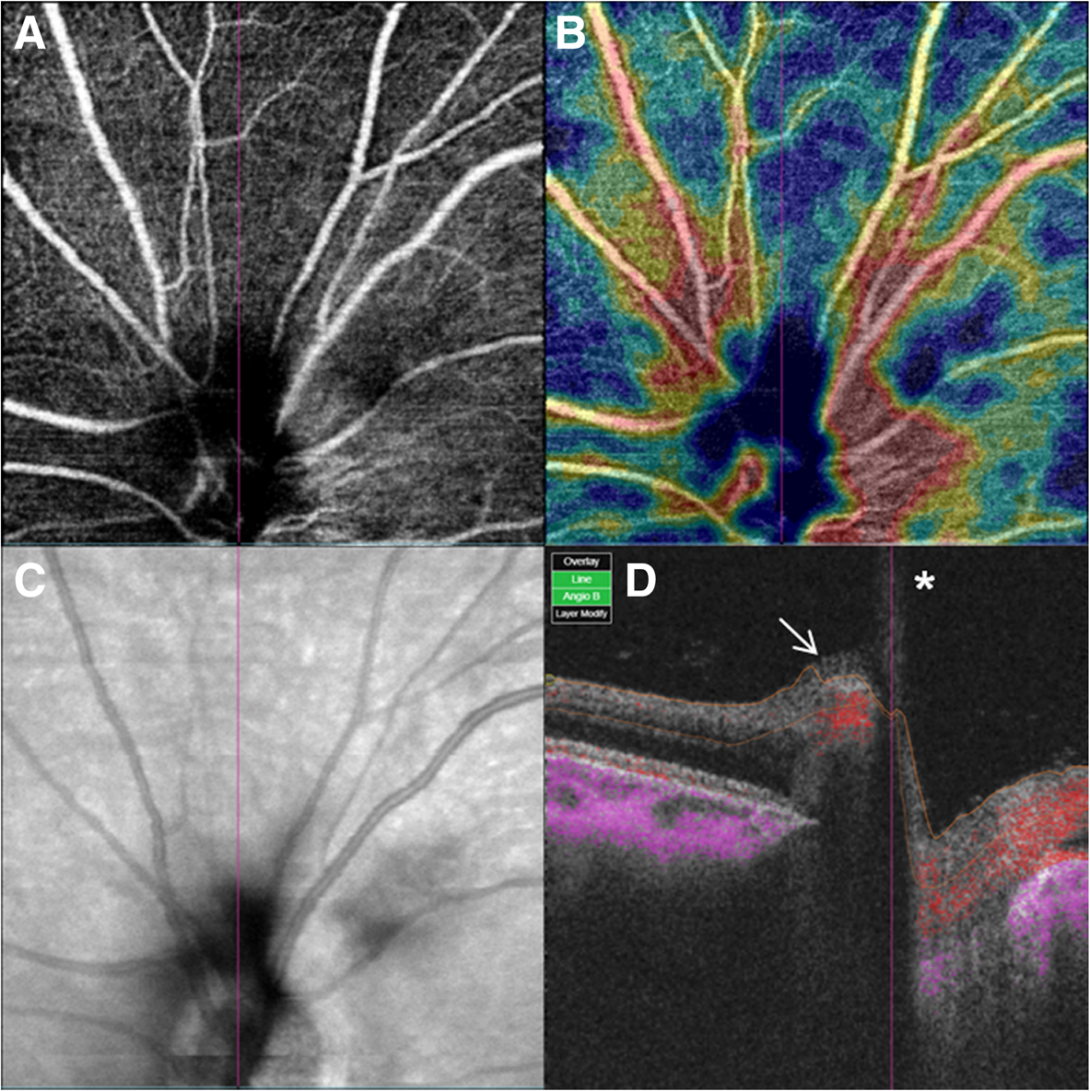
Optical coherence tomography angiography of the optic nerve head of the left eye of the patient taken at 3 weeks after the initial visit. **a** Segmented at the superficial retinal plexus. **b** Color-coded vessel density map. **c** Projection image of optic disc. **d** Cross-sectional OCT angiography. The blood flow was detected from *Bergmeister’s papilla* (arrow), but the blood flow of the persistent hyaloid artery (asterisk) was not confirmed. (Swept Source DRI OCT TRITON. Topcon Medical Systems, Inc. Oakland, USA)

The VH partially decreased 2 weeks later and the BCVA improved to 0.4 logMAR in the left eye. No cause of VH other than the PHA was found (Fig. 1b). Slit-lamp examination demonstrated a *Mittendorf’s dot* located in the inferior nasal quadrant of the posterior lens capsule in the left eye (Fig. 2). Optical coherence tomography (OCT) showed hyporeflective tubular structure of the PHA and elevated tissue structure of the optic nerve (*Bergmeister’s papilla)* (Fig. 3). OCT angiography could not demonstrate the active blood flow of the PHA due to the technical limitation. However, we could find the blood flow in the *Bergmeister’s papilla* (Fig. 4). The VH was spontaneously disappeared after 2 months. No serious disorders have been observed in the left eye during the follow-up period.

## Discussion and conclusions

The PHA is a rare fetal remnant from developmental abnormalities during the seventh month of gestation. The hyaloid artery is a branch of the ophthalmic artery which is a branch of internal carotid artery. It is present in the optic canal and extends from the optic disc to the crystalline lens via the vitreous humor. It branches to form a network of capillaries, the tunica vasculosa lentis, which is prominent at about 9 weeks’ gestation [10, 11]. In healthy fetuses, gradual regression of the hyaloid artery starts at 18 weeks, and by 29 weeks, the hyaloid artery disappears and leaves Cloquet’s canal [12, 13].

The failure of regression of the hyaloid artery is called PHA which may be either partial or complete. The remnant of the anterior portion is called *Mittendorf’s dot* which is located on the inferonasal quadrant of posterior lens capsule and the remnant of the posterior portion is called *Bergmeister’s papilla* which is present at the optic disc usually composed of glial tissue [10, 14]. The entire hyaloid artery which extends from the optic disc to the posterior lens capsule is rare. The patent hyaloid vessels may supply a part of retinal tissue [13]. Hence, the condition is prone to repeated episodes of VH, and contraction of the fibrovascular mass may exert traction on the retina, causing retinal detachment [14].

For diagnosis, slit-lamp examination, fundus examination, fluorescein angiography (FAG), OCT and Doppler ultrasound have been used [3–8, 13, 15]. Gonçalves et al. [4] and Chen et al. [5] used FAG to check patency and leakage of the PHA, while Goncalves et al. reported no blood flow, Chen et al. showed proximal filling. Our patient refused to FAG, so we took OCT angiography which was noninvasive to the patient in order to check the blood flow of the PHA.

Various mechanisms such as the traction force during the rapid eye movement phase of sleep [5], external trauma to the globe [4, 16], and spontaneous development [6] have been reported. In our case, the mechanism of the occurrence of VH from the PHA is unclear. We could not find the presence of posterior vitreous detachment or vitreoretinal traction after VH disappeared. Since the patient had a history of recent excessive weight training, we suppose that VH was caused by rupture of the PHA by elevated blood pressure or physical force due to excessive exercise.

To our knowledge, it is the first report that OCT angiography was used to check blood flow of the PHA. Our OCT angiography could show the blood flow only in the *Bergmeister’s papilla.* Since OCT angiography has low axial resolution, the blood flow in the vitreous cavity is difficult to observe with OCT angiography.Therefore, the blood flow inside the *Bergmeister’s papilla,* which is mainly composed of glial tissue and has no normal blood flow, can be an evidence of patency of the PHA. Another possible reason why the blood flow was observed only in the *Bergmeister’s papilla* is that the blood flow of the PHA was actually only in the proximal part, like the case of Chen et al. [5].

In conclusion, the PHA should be considered as a cause of vitreous hemorrhage in young and healthy patients. The OCT angiography will be a useful noninvasive test to confirm the patency of the PHA.

## Data Availability

All data supporting the findings are contained within the manuscript.

## Abbreviations

BCVA: Best corrected visual acuity
FAG: Fluorescein angiography
OCT: Optical coherence tomography
PHA: Persistent hyaloid artery
VH: Vitreous hemorrhage
